# Anti-tumor Effects of Cisplatin Synergist in Combined Treatment with *Clostridium novyi*-NT Spores Against Hypoxic Microenvironments in a Mouse Model of Cervical Cancer Caused by TC-1 Cell Line

**DOI:** 10.34172/apb.2023.084

**Published:** 2023-05-20

**Authors:** Behrouz Ebadi Sharafabad, Asghar Abdoli, Mohammad Panahi, Lida Abdolmohammadi Khiav, Parisa Jamur, Fatemeh Abedi Jafari, Azita Dilmaghani

**Affiliations:** ^1^Department of Pharmaceutical Biotechnology, Faculty of Pharmacy, Tabriz University of Medical Sciences, Tabriz, Iran.; ^2^Department of Hepatitis and HIV, Pasteur Institute of Iran (IPI), Tehran, Iran.; ^3^Department of Anaerobic Vaccine Research and Production, Specialized Clostridia Research Laboratory, Razi Vaccine and Serum Research Institute, Agricultural Research, Education and Extension Organization, Karaj, Iran.; ^4^Department of Microbiology and Microbial Biotechnology, Faculty of Life Sciences and Biotechnology, Shahid Beheshti University, Tehran, Iran.; ^5^Infectious and Tropical Diseases Research Center, Tabriz University of Medical Sciences, Tabriz, Iran.

**Keywords:** Clostridium novyi-NT, Cisplatin, TC-1 cell line, Cervical cancer, Tumor hypoxia

## Abstract

**Purpose:**

Despite the development of anti-human papillomavirus (HPV) vaccines, cervical cancer is still a common disease in women, especially in developing countries. The presence of a hypoxic microenvironment causes traditional treatments to fail. In this study, we presented a combined treatment method based on the chemotherapeutic agent cisplatin and *Clostridium novyi*-NT spores to treat normoxic and hypoxic areas of the tumor.

**Methods:**

TC-1 Cell line capable of expressing HPV-16 E6/7 oncoproteins was subcutaneously transplanted into female 6-8 week old C57/BL6 mice. The tumor-bearing mice were randomly divided into four groups and treated with different methods after selecting a control group. Group 1: Control without treatment (0.1 mL sterile PBS intratumorally), Group: *C. novyi*-NT (10^7^ *C. novyi*-NT). Group 3: Receives cisplatin intraperitoneally (10 mg/kg). Fourth group: Intratumoral administration of *C. novy*i-NT spores + intraperitoneal cisplatin. Western blot analysis was used to examine the effects of anti-hypoxia treatment and expression of hypoxia-inducible factor 1 (HIF-1) and vascular endothelial growth factor (VEGF) proteins.

**Results:**

The results clearly showed that combined treatment based on *C. novyi*-NT and cisplatin significantly reduced the expression of HIF-1 alpha and VEGF proteins compared to cisplatin alone. At the same time, the amount of necrosis of tumor cells in the combined treatment increased significantly compared to the single treatment and the control. At the same time, the mitotic count decreased significantly.

**Conclusion:**

Our research showed that developing a combined treatment method based on *C. novyi*-NT and cisplatin against HPV-positive cervical cancer could overcome the treatment limitations caused by the existence of hypoxic areas of the tumor.

## Introduction

 It is well known that the human papillomavirus (HPV) is the leading cause of more than 90% of cervical cancers.^1^ The E6 and E7 oncoproteins of high-risk HPV types, particularly HPV16, alter routine host anti-tumor pathways, such as intrinsic apoptosis, and then induce cancer in host cells by generating genomic instability by targeting two host tumor suppressors, p53 and the retinoblastoma protein (pRb).^2,3^ Despite developing prevention methods such as vaccination of adolescent girls against high-risk strains of HPV and modern diagnostic procedures in developed countries, in poor and less developed countries, 500 000 new cases of cervical cancer and more than 300 000 deaths are reported due to this disease.^4-6^ Today, various treatment regimens based on surgery, chemotherapy, and Radiotherapy are combined to treat cervical cancer.^7^ However, there are still challenges to treatment success.^8^ An in-depth understanding of HPV-related malignancies and treatment failure factors is essential to provide successful treatment regimens.

 The fact that solid cervical tumors have hypoxic areas with oxygen concentrations below 1.5% has been proven.^9^ Hypoxia is an adverse prognostic factor that suppresses the host’s immune system and severely reduces the effectiveness of various treatments, including Radiotherapy and chemotherapy.^10,11^ It has recently been shown that under hypoxic conditions, HPV-infected tumor cells, by being in a reversible proliferative stasis state, drastically reduce E6/E7 expression and cause the cell to escape immunity.^12^ If tumor cells are in the proper oxygen delivery state, the expression of E6/E7 oncoproteins resumes rapidly and, after treatment, acts as an abundant source of oncoprotein expression.^12,13^ This complex interaction, which can eventually cause the tumor cell to escape from the senescent, is mediated through the phosphoinositide 3-kinase/AKT/mammalian target of rapamycin (mTOR) signaling pathway in hypoxic conditions, despite decreased E6/E7 oncoprotein expression.^13-16^ Hypoxia-inducible factor 1-alpha (HIF-1α) acts as a significant protein in the adaptation of cancer cells to hypoxic conditions, and its role in the angiogenesis and development of various cancers has been discussed.^17,18^ By stabilizing HIF-1α in hypoxic conditions in cervical cancer, regulated in development and DNA damage responses 1 is activated and inhibits mTORC1.^15,19,20^ As a result, despite AKT-mediated inhibition of E6/E7, hypoxic HPV-positive cancer cells survive the aging process.^13^ Thus, targeting tumor cells in hypoxic areas can be vital to a successful treatment regimen.

 Cisplatin is one of the most common chemotherapy drugs prescribed as a first-line treatment for many cancers.^21^ This substance induces intrinsic cell death by creating irreversible bindings to the DNA of tumor cells.^22^ However, research has shown that the presence of a hypoxic microenvironment in the tumor, through decreased expression of anti-tumor protein p53,^23^ increased cancer stemness,^24^ regulation of non-coding RNAs,^25^ generation of reactive oxygen species (ROS),^26^ increased exosome secretion,^27^ loss of mismatch repair,^28^ cyclophilin overexpression,^29^ Surviving overexpression,^30^ and increased glucose transporter 1 (GLUT1) expression^31^ may increase the resistance of hypoxic tumor cells to this treatment. Therefore, offering complementary therapies based on anti-hypoxia agents, which target the expression and stability of the HIF-1α axial protein that plays a crucial role in inducing this resistance,^32^ is considered a promising factor in increasing therapeutic efficacy.

 Today, various strategies have been developed to treat all types of solid tumors, including surgery, chemotherapy, radiotherapy, targeted therapy, immunotherapy, combination therapy, and extracellular vesicle therapy.^33-35^ More than half of cancer patients consider Radiotherapy as part of their treatment plan. Despite the benefits of this treatment modality, the development of various physical complications and, most importantly, the susceptibility of the patient’s normal cells to carcinogenesis from radiation exposure appears to be a significant challenge.^36^ In the context of immunotherapy, immune checkpoint inhibition is considered standard treatment, but the most significant concern is for cold cancers, which are unlikely to respond to such therapy.^37^ Some medicines are inefficient because not every tumor can be removed by surgery or because it is resistant to chemotherapy and radiotherapy. Chemotherapy decreased drug penetration into neighboring tumor cells in the weakly vascularized area, environmental toxicity and reduced immunity, which faces some clinical problems after treatment.^38^ The existence of these challenges in treatment, driven mainly by the disturbances in tumor physiology and the presence of hypoxic areas in all types of solid tumors, led researchers to revive the method of using anaerobic bacteria at the beginning of the 20th century, because after injecting these bacteria into the tumor, they grow selectively in the hypoxic areas of the tumor and can cause its destruction.^39^ The existence of these challenges in treatment, caused mainly by the disturbances in tumor physiology and the presence of hypoxic areas in all types of solid tumors, led researchers to revive the method of using anaerobic bacteria at the beginning of the 20th century, because after injecting these bacteria into the tumor, they grow selectively in the hypoxic areas of the tumor and can cause its destruction.^39^

 One of the most attractive ways to overcome the resistance of cancer cells in hypoxic areas of solid tumors to standard therapies, which have been vigorously pursued over the last two decades, is to use the spores of a non-lethal type of *Clostridium novyi.*^40^ After injection into the tumor, spores of *C. novyi*-NT migrate to hypoxic areas of the cancer, and due to the hypoxic nature of these areas, begin to germinate and cause cells to lysis in this area.^41^ The nature of the therapeutic function of this agent is not fully understood,^42^ but it appears that this bacterium induces a robust immune response from the host.^43^ On the other hand, by secreting several extracellular proteins lysing the lipid structure of the cell membrane, including phospholipase C (PLC) (NT01CX0979), it causes the destruction of tumor cells in the hypoxic areas.^44^

 In this study, for the first time, a combined treatment strategy based on cisplatin and *C. novyi*-NT spores is presented in a mouse model for HPV-related cervical cancer and the effects of this type of treatment on resistance factors in hypoxic tumor areas are shown. Understanding these effects could pave the way for combination therapies to make cisplatin more effective against HPV-related cancers.

## Materials and Methods

###  Preparation of cell culture and Clostridium novyi-NT spores

 To cause HPV-Associated cervical cancer in a mouse model, the TC-1 cell line with the ability to express HPV-16 E6/7 oncoproteins^45^ was prepared by the National Cell Bank of Iran affiliated with Pasteur Institute (Tehran, Iran). TC-1 cell line was suspended in RPMI-1640 (Sigma, USA) medium with 10% fetal bovine serum (FBS) (Gibco, USA), 1% penicillin-streptomycin (Sigma, USA), 25 mM HEPES (Sigma, USA), 1% glutamine (Sigma, USA) and incubated at 37 °C and 5% CO_2_. We have previously been able to generate spores without lethal genes from the wild *C. novyi* type B strain.^46^ After processing, *C. novyi*-NT spores were packaged in lyophilized tablets containing 10^7^ and stored at -20 °C for future use.

###  Animal study design and creation of a mouse model of HPV-associated cervical cancer

 Thirty female 6-8 weeks old C57/BL6 mice weighing 18 to 22 g were obtained from Pasteur Institute (Iran, Karaj) and fed for seven days according to laboratory standards. The animals were then randomly divided into five groups and six mice in each group (group = 5 and n = 6). One group was randomly chosen as a non-tumor and healthy group, and the other four groups were selected to challenge the tumor. To cause HPV-Associated cervical cancer, one million TC-1 cells were suspended in 0.2 mL of phosphate-buffered saline (PBS) (Sigma, USA) and injected subcutaneously into the right flank of each mouse. During this period, the mice were monitored daily, and with the appearance of palpable tumors, the size of the tumors was measured using a caliper. The volume of the tumors was determined using the standard formula (longest diameter of the tumor)×(shortest diameter)^2^× 0.5.

###  Treatment with Clostridium novyi-NT spores and cisplatin 

 Cisplatin (CAS 15663-27-1) was purchased from sigma Aldrich (Sigma, USA). To evaluate the therapeutic effects of cisplatin chemotherapy alone or in combination with the anti-tumor effects of *C. novyi*-NT spores, after reaching the volume of mouse tumors in the range of 300 to 500 mm^3^, the following was performed; Group 1: They did not receive any treatment and were injected with 0.1 mL of sterile PBS intratumoral. Group 2: A lyophilized tablet containing 10^7^ *C. novyi*-NT spores was suspended in 0.1 mL of sterile PBS and injected intra-tumor into different tumor parts. Group 3: 10 mg/kg cisplatin^47^ was dissolved in 0.1 mL sterile PBS and injected intraperitoneally into each mouse. Group 4: This group is a combination therapy group and received 10^7^ *C. novyi*-NT spores suspended in 0.1 ml PBS on day 0. Subsequently, 8 hours later, they received 10 mg/kg cisplatin dissolved in 0.1 mL PBS intraperitoneally. According to our previous study,^46^ the 15th day after the start of treatment was chosen as the day of the end of treatment; on this day, all animals received 30 μL of anesthetic solution with the following characteristics: Ketamine 10% (100 mg/mL; Medistar, Ascheberg, Germany) and xylazine 2% (20 mg/mL; Riemser, Greifswald, Germany) were combined in a single insulin syringe (2 parts ketamine and 1 part xylazine)^48^ and After ensuring deep anesthesia of the animals, a blood sample was taken through the heart muscle and maintained at 10% using EDTA (ethylene diamine tetraacetic acid) as an anticoagulant. The animals were then sacrificed through the displacement of the neck vertebra. Tumor tissue was then carefully isolated and kept at -70 °C for gene analysis and protein expression and in 10% formalin solution for histopathological examination.

###  RNA extraction and cDNA synthesis

 Total RNA was extracted from 100 mg of cervical cancer tissue isolated from each mouse and used RNA Extraction kit (Thermo Fisher Scientific, USA) according to the manufacturer’s instructions. The purification of total RNA was evaluated by NanoDrop ND-1000 (NanoDrop, USA) spectrophotometer. Extracted product was tested on a 2% agarose gel to check RNA integrity. 500 µg of extracted RNA was used for cDNA synthesis by random hexamer primer using a reverse transcription kit (Biotech Rabbit, Germany) according to the manufacturer’s instructions.

###  Real-time polymerase chain reaction (RT-PCR) analysis 

 The expression level of the GLUT1 and PLC genes was determined by RT-PCR. 2 µL of syntonies cDNA was subjected to PCR cycle with SYBR Green 2x Master Mix (Amplicon, Denmark). PCR conditions included pre-denaturation at 95 °C for 15 minutes, denaturation at 95 °C for 30 seconds, and denaturation at 60 °C for 45 seconds for 40 cycles. Glyceraldehyde 3-phosphate dehydrogenase (GAPDH) was used as a reaction internal reference. All the samples were determined 3 times. The special primers were as follows: GLUT1, forward: 5’- GAGAACCGGGCCAAGAGTG-3’ and reverse 5’- TTCTTCTCCCGCATCATCTG-3’; PLC, forward 5’- GGAGCATCAAGTAAAGCGTA-3’ and reverse 5’- CATTCGGATCATAATCAGGA-3’; GAPDH, forward 5’-GCCAAAGGGTCATCATCTC-3’ and reverse 5’-GTAGAGGCAGGGATGATGTT-3’. All primers were designed by Oligo 7 software and synthesized by Metabion Company (Germany). The target mRNA value was measured by comparison with the control sample, then the comparison period threshold (ΔΔCt) method was used for calculation.

###  Western blot analysis

 Western blot analyses were performed as previously described with some modifications.^49^ The lysates were removed by centrifugation at 14 000 rpm for 20 minutes at 4 °C. According to the manufacturer’s instructions, the BCA protein Quantification kit determined the protein concentration of exosome lysates. The exosome lysates were mixed with a 2X Laemmli sample buffer equal to volume. Lysates (15 μg) were then subjected to SDS-PAGE after a 5 minutes boiling and subsequently transferred to a 0.2 μm immune-Blot^TM^ polyvinylidene difluoride (PVDF) membrane (Bio-Rad Laboratories, CA, USA). The membranes were then blocked with 5% BSA (Cat No: A-7888; Sigma Aldrich, MO, USA) in 0.1% Tween 20 for 1 hour. Then, the membranes were incubated with Anti-HIF-1 alpha (Abcam), Anti- Vascular endothelial growth factor (VEGF) (Abcam), and anti-beta actin-loading control antibodies (Abcam) for one h at room temperature. Subsequently, membranes were washed thrice with TBST and incubated with goat anti-rabbit IgG H&L (HRP) (Abcam) secondary antibodies. The membranes were then incubated with enhanced chemiluminescence for 1–2 minutes. Protein expression was normalized to β-actin. Densitometry of protein bands was performed using the gel analyzer Version 2010a software (NIH, USA), such that the percentage area under the curve of each band was divided by the percentage area under the curve of its corresponding actin band, and then calculated values were compared between groups as described previously.^49^

###  Reactive oxygen species analysis

 ROS1/ROS ELISA Kit (LifeSpan BioSciences, USA) was used to measure the number of ROS based on the Sandwich ELISA method. Tumor tissues were washed in PBS to remove excess blood. Next, the tissues were weighed before homogenization. The tissues were minced and homogenized in 10 ml PBS with a glass homogenizer on ice. All reagents, working standards, and samples were prepared according to the kit instructions and placed at room temperature for 20 minutes. 100 µL of sample and standard were added to each well and incubated for 2 hours at 37 °C. The plate was emptied, and 100 µL of Detection Reagent A was added and incubated for 1 hour at 37 °C. The plate was opened and washed thrice with 400 µL of the wash solution in the plate kit. 100 µL of Detection Reagent B was added and incubated for 30 minutes at 37 °C. The plate was emptied and washed thrice with 400 µL of the wash solution in the plate kit. 90 mL of substrate solution was added to each well and incubated for 20 minutes in the dark at 37 °C. Finally, 50 µL of Stop solution was added to each well. Using an ELISA device, the amount of light absorption was read at 450 nm, and based on the amount of light absorption, the standard curve was drawn, and based on the slope of the line and the width of the beginning, and the amount of ROS concentration in each well was measured.

###  Histopathological studies

 Tumor tissue was fixed in 10% formalin and dehydrated through graded ethanol (70%, 90%, 96%, and 100%). Then, the tissues were paraffin-embedded, and 5μm sections were taken from the tissue by microtome. So, the slides were stained with hematoxylin and eosin dyes and Giemsa staining according to routine laboratory protocols. And then, for the histology of tumors, conventional hematoxylin and eosin (H&E) staining and light microscopy were used. Formula calculated the relative necrotic area (%) of tumor tissues: Relative necrotic area (%) = Necrotic area in tumor section/Total area of tumor section × 100.^50^

###  Statistical analysis

 All data for this experiment have been presented as mean ± SD. We used GraphPad Prism 9 software for statistical analysis, including a one-way analysis of variance (ANOVA) and a *t* test, and selected 0.05 as statistically significant.

## Results and Discussion

###  Clostridium novyi-NT spores combined with cisplatin showed a synergistic anti-tumor effect in TC-1 mice models

**Figure 1:**
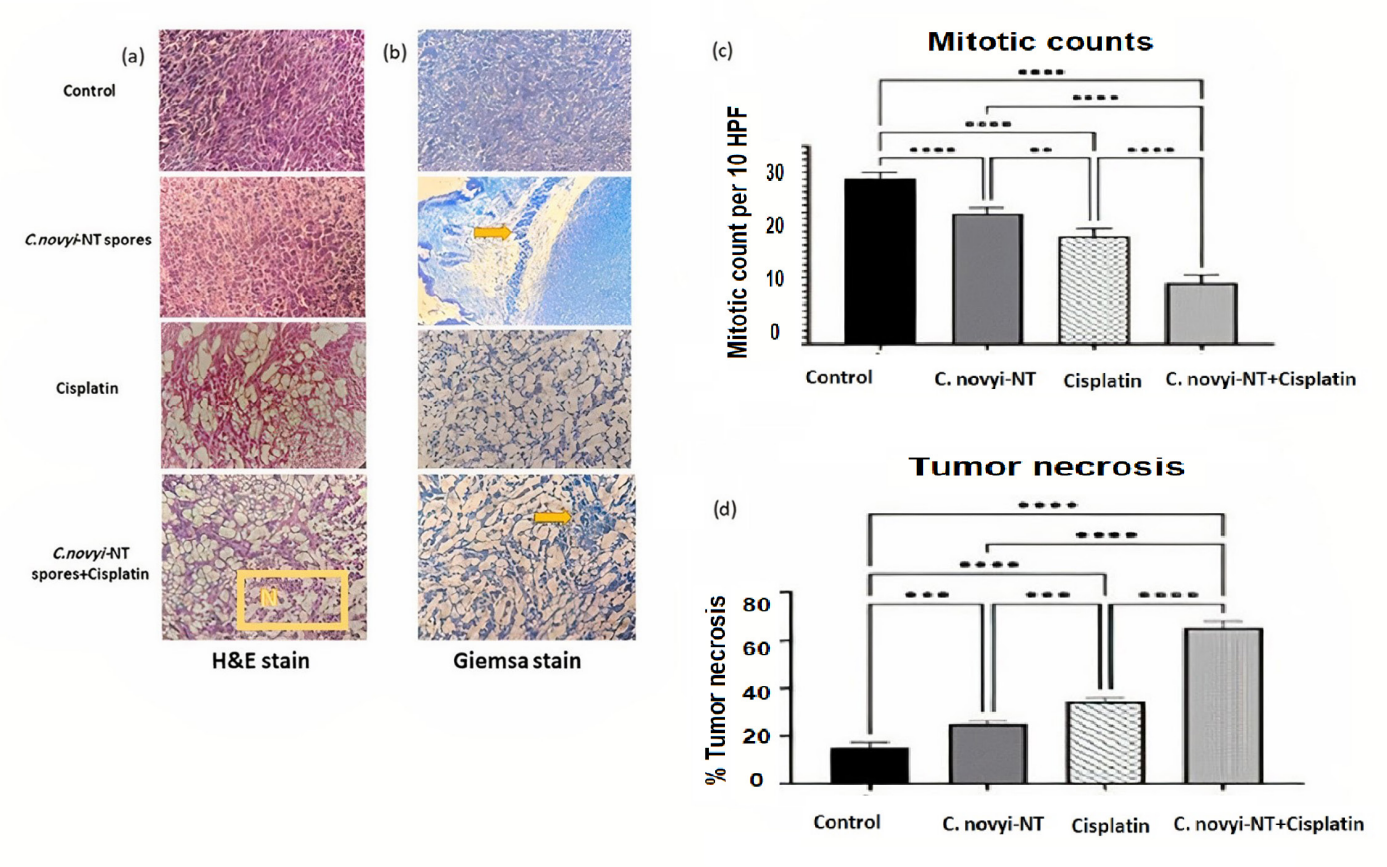
Histopathological observations of the therapeutic combination of *Clostridium novyi*-NT spores and cisplatin in TC-1 tumor-bearing mice: (a) The results of H&E staining show that extensive necrosis has occurred in the tissue of tumor mice receiving the combination treatment (yellow box: extensive necrosis of tumor tissue). (b) Giemsa staining, confirming the germination of *Clostridium novyi*-NT bacteria spores in treatment groups receiving spores (yellow arrow: bacterial accumulation). (c) The comparison of the mitotic count of the tested groups shows that the amount of this factor is statistically significantly lower compared to the control group and the group receiving cisplatin. (d) Extensive tumor necrosis of the tumor tissue in the group receiving the combined treatment, the amount of which has increased statistically significantly compared to the other groups and the control group. (* *P* < 0.05, ** *P* < 0.005, *** *P* = 0.0001, **** *P* < 0.0001)

 The tumor tissues stained by the H&E technique were carefully examined to understand the double anti-tumor function of the combined treatment based on *C. novyi*-NT sporesand the cisplatin (Figure 1a). The detailed examination showed that the percentage of necrosis in the treatment group that received cisplatin and bacteria compared to the group that received only cisplatin increased statistically significantly (*P* < 0.0001) (Figure 1c). Also, detailed microscopic examinations showed that the mitotic count, as an indicator of tumor malignancy, in the group receiving combined treatment was statistically significantly lower compared to the control group and the group treated only with cisplatin (*P* < 0.0001) (Figure 1d).

###  Effects of C. novyi-NT spores and cisplatin on GLUT 1 mRNA expression level in mice with cervical cancer and qPCR for detecting C. novyi-NT 

**Figure 2:**
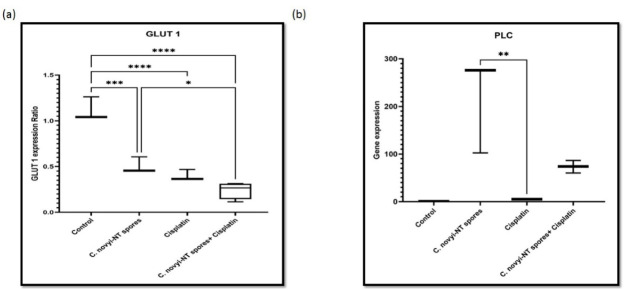
Effects of *Clostridium novyi*-NT spores and cisplatin on the expression level of PLC and GLUT 1 in mice with cervical cancer. GAPDH was used as a housekeeping gene. The RT-qPCR results showed statistically significant differences in the mRNA expression levels of (a) GLUT 1 and (b) PLC in different treatment groups of mice compared to the control. Data demonstrate the mean ± SD of three independent experiments. (* *P* < 0.05, ** *P* < 0.005, *** *P* = 0.0001, **** *P* < 0.0001)

 Glucose transporter-1 has been suggested as a prognostic factor in various cancers associated with treatment resistance and immune evasion.^51^ In a study by Kim et al, with the aim of intending to investigate the predictive effect of GLUT1 in cervical cancer, they analyzed the data of 298 patients. They showed that high expression of GLUT1 with old age, squamous cell carcinoma, high tumor stage, metastasis to pelvic lymph nodes, and low hysterectomy rates are associated. Finally, they concluded that GLUT1 expression and HPV16 subtype might have independent prognostic value in cervical cancer. GLUT1-mediated immune modulation may be one of the crucial reasons for treatment failure, especially in HPV16 positive group.^52^ As a result, it can be concluded that treatments that modulate the expression of GLUT1 will have promising efficacy. The qPCR technique was employed to study the effect of *C. novyi*-NT spores and cisplatin treating cervical cancer in mice. The GAPDH gene was applied as a housekeeping gene (Figure 2), showing the expression level of GLUT 1 and *C. novyi*-NT PLC (Gene: NT01CX_0979) in different groups of mice. Results show that the GLUT 1 expression was significantly reduced in *C. novyi*-NT spores compared to control (*P* = 0.0001), the combination of C. *novyi*-NT spores and cisplatin compared to *C. novyi*-NT spores (*P* < 0.05), cisplatin compared to control and variety of *C. novyi*-NT spores and cisplatin compared to control (*P* < 0.0001). *C. novyi*-NT PLC (Gene: NT01CX_0979) was considered a factor confirming the presence and germination of *C. novyi*-NT spores. As expected, the expression level of this gene was zero in the control group and the group that received only cisplatin, as the PLC expression levels were significantly increased in cisplatin compared to control and cisplatin compared to *C. novyi*-NT spores (*P* < 0.005) and was seen the reduced level of expression in the combination of *C. novyi*-NT spores and cisplatin compared to cisplatin (*P* < 0.05) (Figure 2).

###  Effects of C. novyi-NT spores and cisplatin on HIF-1α and VEGF proteins expression level in mice with cervical cancer 

**Figure 3:**
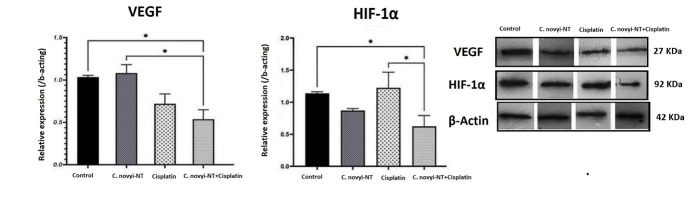
Western blotting representative of HIF-1α and VEGF of different treatment groups, β-actin was used as a normalizer. A statistically significant decrease in the expression of both proteins is evident in the group receiving the combined treatment of *Clostridium novyi*-NT spores and cisplatin compared to the control group. All the experiments were performed in triplicates. (* *P* < 0.05)

 Hypoxia-inducible factors have been identified in the hypoxic tumor microenvironment as essential transcription factors that regulate the expression of many of genes related to angiogenesis, metastasis, cell proliferation, and resistance to chemotherapy and radiotherapy. HIF-1α promotes cancer cell proliferation, and VEGF induces vascular endothelial cell division to promote tumor growth. High expression of HIF-1α and VEGF in cervical cancer tissues is associated with clinical stage, pathological grade and lymph node metastasis.^53^ The effect of *C. novyi*-NT spores and cisplatin on protein expression level was examined by western blot and used anti-beta actin-loading by control antibodies. There were statistically significant differences in the protein expression levels of HIF-1α and VEGF in different groups of mice. Figure 3 shows protein expression levels in all groups of mice. Results show that the HIF-1α protein expression significantly reduces in the combination of *C. novyi*-NT spores and cisplatin group compared to control (*P* < 0.05), the combination of *C. novyi*-NT spores and cisplatin group compared to cisplatin (*P* < 0.05). The VEGF protein expression significantly reduces in the combination of *C. novyi*-NT spores and cisplatin group compared to control (*P* < 0.05), the combination of *C. novyi*-NT spores and cisplatin group compared to *C. novyi*-NT spores (*P* < 0.05).

###  Effects of C. novyi-NT spores and cisplatin on ROS in mice with cervical cancer

**Figure 4:**
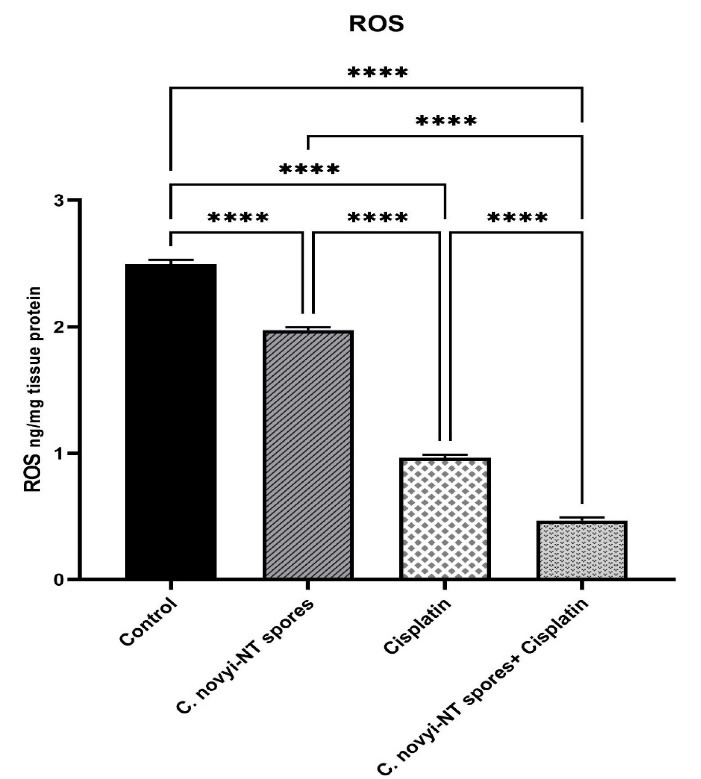
Comparison of intracellular levels of ROS in different treatment groups: The results showed that the number of ROS was significantly reduced in the group receiving cisplatin along with *Clostridium novyi*-NT spores compared to the group receiving cisplatin and *Clostridium novyi*-NT spores alone and compared to the control group (*P* = 0.0001)

 In several types of cancer, ROS are widely associated with carcinogenesis and cancer progression.^54^ To investigate the effect of *C. novyi*-NT spores and cisplatin on the changes related to Reactive oxygen species (ROS), it was performed according to the protocol of ROS1 / ROS ELISA Kit (LifeSpan BioSciences, USA). The results showed that the number of ROS was significantly reduced in the group receiving cisplatin along with *C. novyi*-NT spores compared to the group receiving cisplatin and *C. novyi*-NT spores alone and compared to the control group (*P* = 0.0001) (Figure 4).

 It has been well established that the most important cause of cervical cancer is a chronic infection caused by high-risk types of HPV.^55^ These viruses cause genomic instability and cancer by deactivating suppressor proteins p53 and pRb through oncoproteins E6 and E7. Despite the development of prevention methods for this malignancy based on vaccination against high-risk types of HPV, the infection rate is still high in poor and developing countries, and as a result, there is a need to develop new treatment approaches.^56^ One of the essential factors that strongly affect the success of conventional treatments of this disease is the presence of hypoxic micro-regions in this type of tumor. Therefore, it can be said that hypoxia is the most critical factor in the failure of conventional treatments based on chemotherapy and radiotherapy.^57^

 The most important factor that causes cancer cells to adapt to hypoxic conditions is HIF-1α. This transcription factor, as the central axis of cell adaptation to hypoxic conditions, by regulating more than 100 genes, mediates the requirements for the continued abnormal proliferation of tumor cells, abnormal metabolisms, metastasis, and resistance to therapeutic agents.^53,58^ As a result of the stabilization of this protein, VEGF expression is induced, which is a potential factor in the induction of tumor angiogenesis and also causes the expression of the glucose transporter gene and thus increases glycolysis.^59^

 One of the compounds widely used in treating various malignancies, including cervical cancer, is cisplatin.^60^ This combination has significant effects on normoxic tumors, but the hypoxic condition of the tumor is the main reason for the failure of this treatment.^21^ Research has shown that cisplatin causes the death of tumor cells through upregulating of the p53 suppressor protein.^22^ However, in the hypoxic conditions, HIF-1α strongly suppresses the level of p53.^61^ As a result, the resistance to this treatment increases. At the same time, as oxygen decreases in the hypoxic conditions of the tumor, the leakage of electrons from the electron transport chain occurs, which is the reason for the reduction of the electron flow through the mitochondrial complex of the electron transport chain. This process produces ROS that can enhance mitochondrial fragmentation and further reduce the expression of p-Drp1 and Mfn1, resulting in increased resistance to cisplatin. Therefore, providing combined treatment regimens based on cisplatin and tumor anti-hypoxic agents can promise to overcome the therapeutic limitations of cisplatin and significantly strengthen its effectiveness in hypoxic conditions.^21,62^

 The hypoxic condition of the tumor creates a suitable environment for the germination and growth of *C. novyi*-NT spores.^63,64^ We and others have shown that this species, simultaneously germination and growing in hypoxic regions of tumor types, has caused extensive regression in various tumors in rodent models.^46,65,66^ Due to the unclear nature of this function, we tried to present a combined treatment method based on cisplatin and *C. novyi*-NT spores in the HPV-related cervical cancer model. Our goal was to increase the therapeutic efficiency and overcome the resistance of cisplatin treatment in hypoxic conditions.

 This study showed that the expression level of HIF-1α and VEGF proteins decreased in the group of tumor mice that received the combined treatment, in a statistically significant way, compared to the control group. By examining the aberrant expression patterns of 3352 differentially expressed genes in 306 cervical cancer samples, Xu et al concluded that the HIF-1 signaling pathway related to TFRC might play an important role in cervical cancer.^67^ Conversely, Liu et al showed that HIF-1α and VEGF could be considered a parameter in evaluating the progress, metastasis, and prognosis of HPV-related cancers.^68^

 Various studies have shown that VEGF plays a significant role in angiogenesis and cancer development. On the other hand, abnormal expression of GLUT 1, as a downstream gene of HIF-1α, in HPV-positive head and neck cancer tumors increases the probability of invasion and metastasis. Therefore, it can be said that the decrease in the expression of this gene is a positive sign of the effectiveness of various therapeutic strategies. Fortunately, our recent study also showed that the expression level of GLUT 1 was significantly reduced in the group that received the combined treatment of *C. novyi*-NT spores and cisplatin.

 Transmission electron microscopy and atomic force microscopy revealed that *C. novyi*-NT spores are surrounded by an amorphous layer interwoven with parasporal honeycomb layers sequentially dissolved during germination. Vegetative cells most of these spore-specific genes encode spore coat proteins or proteins with redox activity, which could aid germination by scavenging ROS.^44,69^ As mentioned earlier, in hypoxic tumors, the reduction in oxygen consumption causes a decrease in electron flow through the mitochondrial complex of the electron transport chain.^26^ This leads to the leakage of electrons from the electron transport chain, resulting in excessive production of ROS, which in turn increases cisplatin resistance. In various tumors, the hypoxic tumor microenvironment induces ROS production, which increases mitochondrial fission and, thus, cisplatin resistance by downregulating the expression of p-Drp1 (Ser637) and Mfn1.^62^ Our study showed that the number of ROS in the group receiving combined treatment was significantly reduced compared to the control group.

 The molecular mechanisms involved in the anti-tumor function of *C. novyi*-NT have not been fully established. However, it seems to be related to the destructive properties of the enzymes it secures the induction of host immunity by the secretion of large amounts of cytokines.^40,70^ In 2006, Bettegowda et al determined the genomic sequence of *C. novyi*-NT spores. They showed that *C. novyi*-NT could affect the structure of the lipid layers of the host cell wall by secreting various proteins such as PLC (NT01CX0979), which, in addition to having a direct effect on tumor cell lysis, Activation of host antitumor immune responses is also stimulated.^44^

 In this study, we measured the germination of *C. novyi*-NT spores by measuring the amplification of the PLC gene (NT01CX0979), along with the use of Giemsa stain. The results showed that, as expected, the expression level of this gene was zero in the control group and the group that received only cisplatin. As the PLC expression levels were significantly increased in cisplatin compared to control and cisplatin compared to *C. novyi*-NT spores and seen the reduced level of expression in the combination of *C. novyi*-NT spores and cisplatin compared to cisplatin.

 The results clearly showed that using a combined treatment regimen based on *C. novyi*-NT spores and cisplatin can overcome the therapeutic limitations of cisplatin chemotherapy in tumors with hypoxic areas in cervical cancer. It also increases treatment efficiency and is considered a favorable option in developing combined treatment methods for solid hypoxic tumors.

## Conclusion

 Our research showed that providing a combined treatment method based on *C. novyi*-NT spores and cisplatin can overcome the limitations and therapeutic resistance caused by hypoxic microenvironments in HPV-positive cervical cancers. However, the mechanism of this phenomenon is not completely clear, but by overcoming the existing limitations, this method has a positive perspective in developing effective anti-tumor hypoxia treatment methods.

## Acknowledgments

 We sincerely thank Iran National Science Foundation (INSF) for its financial support. We thank Tabriz University of Medical Sciences and the Pasteur Institute of Iran for supporting this research.

## Competing Interests

 The authors declare that they have no direct or indirect conflict of interest.

## Ethical Approval

 This study was reviewed and approved by the Research and Technology Vice-Chancellor of Tabriz University of Medical Sciences (IR.TBZMED.VCR.REC.1398.434). Also, all animal work was under the standards of the Iran National Committee for Ethics in Biomedical Research and was performed under the supervision of the Ethics Committee in Animal Research of the Pasteur Institute of Iran.
